# Injuries to the Eyeball

**Published:** 1878-04

**Authors:** 


### The Bistoury.

ELMIRA, N. Y., APRIL, 1878.

The BISTOURY is published Quarterly, upon the 1st of April, July, October and January, at Fifty Cents a year in advance.

### INJURIES TO THE EYEBALL.

Now and then a patient comes to us in great alarm, by reason of a ruptured vessel under the conjunctiva, giving the white portion of the eye a blood-red appearance, often throughout its entire extent; the clear, bright cornea looking like a crystal set in a piece of raw meat. Such ruptures occur from a sudden, violent sneezing or coughing, and are not apt to do any harm to the eye. In a few days the blood will disappear, through absorption, and leave the eye as clear as before.

Sometimes such an effusion of blood may occur from a blow upon the eye, from a whip, ball, or piece of wood striking the eye. If no considerable pain is the result, the simple "blood-shot" appearance of the eye need give no alarm; but, where pain follows the blow, and hot tears run over the cheek, and the eye shuns the light, no delay should be indulged in, but an ophthalmic surgeon sought at once, who, by aid of the ophthalmoscope will examine the inside of the eye, and so ascertain what injury has been done.

Pain, after blows upon the ball, particularly when it continues for more than a few hours, is almost certain proof that some serious injury has been done the delicate internal structure of the eye. It points to rupture of the iris, dislocation of the lens, or injury to the retina, or nerve, in all or any of which conditions, prompt treatment must be had to save the injured organ from complete loss of function.

Some dangerous playthings are permitted among children that are fruitful causes of blindness. The very worst that has come to our notice consists of a forked stick, to the prongs of which are attached two strong elastic bands, terminating in a leather sling, for holding a marble, pebble, buck-shot or other projectile. The missile is thrown with such violence as to endanger life as well as vision. While we write, a young lad in the next room, is suffering from the loss of an eye, injured by one of these dangerous implements. The explosion of gun-caps, with stone, hammer, or upon the end of an iron rod, has ruined the eyes of very many children. It would seem that the particles of copper flying from the exploded caps, seek the eye at once, and almost invariably find it. Bows and arrows, we are sorry to see, are coming in use again, as the Spring days are tempting the children to out-door sports. We have known about as many children's eyes hit with the arrows from this implement of barbarian warfare, as "bull's eyes," and for this reason earnestly advocate its banishment from the possession of our youthful sportsmen.

If a blow is received upon the eyeball with any sharp instrument, like a piece of glass, slate, a dart, or other cutting or piercing point, *immediate* treatment is necessary to prevent the *aqueous humor* from escaping and carrying the *iris* into the wound so causing a deformed, if not a lost, eye.

In all accidents of this sort no "eye waters" should be used,—nearly all patent nostrums of this character contain sugar of lead, a fatal drug to injuries of the cornea. Bind up the eye with a cloth saturated with cold water,—nothing else. Avoid arnica, and poultices of all kinds. Seek competent advice early, and so avoid an incalculable amount of trouble, and perhaps a lost eye.

